# Development and validation of automated methods for COVID-19 PCR Master Mix preparation

**DOI:** 10.1016/j.slast.2024.100195

**Published:** 2024-10

**Authors:** Giorgio Fedele, Graham Hill, Amelia Sweetford, Suki Lee, Bobby Yau, Domenico R. Caputo, Denise Grovewood, Rowda Dahir, Paula Esquivias Ruiz-Dana, Anika Wisniewska, Anna Di Biase, Miles Gibson, Benita Percival, Stefan Grujic, Donald P. Fraser

**Affiliations:** a Department of Biology, Ecology and Earth Science, University of Calabria, Arcavacata, Italy; b Animal and Plant Health Agency, Weybridge, United Kingdom; c Department of Biomolecular Health Sciences, Faculty of Veterinary Medicine, Utrecht University, Utrecht, The Netherlands; d University of Huddersfield, Huddersfield, United Kingdom; e SPT Labtech, Melbourn Science Park, United Kingdom; f University of Cambridge, Cambridge, United Kingdom; g Health Protection Operations, UKHSA, United Kingdom; h Omix Ltd, Birmingham, United Kingdom; i Independent Scholar

**Keywords:** Automation, High-throughput, Molecular diagnostics, COVID-19, PCR, Liquid handling

## Abstract

Polymerase chain reaction (PCR)-based assays were widely deployed during the SARS-CoV-2 pandemic for population-scale testing. High-throughput molecular diagnostic laboratories required a high degree of process automation to cope with huge testing demands, fast turnaround times, and quality requirements. However, process developers and optimizers often neglected the critical step of preparing a PCR Master Mix. The construction of PCR Master Mix depends on operator skill during the manual pipetting of reagents. Manual procedures introduce variation, inconsistency, wastage, and potentially risks data integrity. To address this, we developed a liquid-handler-based solution for automated, traceable, and compliant PCR Master Mix preparation. Here, we show that a fully automated PCR Master Mix protocol can replace manual pipetting, even in a diagnostic environment, without affecting accuracy or precision. Ultimately, this method eliminated operator-induced wastage and improved the consistency of the quality of results.

## 1 Introduction

Nucleic acid amplification tests (NAATs) utilize polymerase chain reaction (PCR) [1], [2] as the underlying technology to detect the presence or absence of specific nucleic acid sequences. Such tests were widely employed at large scales around the world during the COVID-19 pandemic to diagnose symptomatic and asymptomatic patients, and track the spread of the SARS-CoV-2 virus. PCR-based assays specifically amplify very small quantities of analyte to measurable levels and, when compared to other viral detection technologies, such as antigen testing, are recognized as the gold standard in terms of sensitivity and specificity for COVID-19 diagnostics [3].

A critical stage in conducting a PCR reaction is the preparation of the PCR Master Mix (MMIX). Typical 1-step MMIX for the detection of RNA contain reverse transcriptase (RT) enzymes, DNA polymerase enzymes, free deoxynucleotide triphosphates (dNTPs), TaqMan probes, and primers suspended in a buffer containing Mg${}^{2+}$ in precise concentrations optimized for the application. These reagents might be supplied separately, or partly combined in pre-prepared concentrations as a kit for ease of use. Laboratory personnel follow protocols detailed in instructions for use (IFU) from the kit inserts or standard operating procedures (SOPs) to prepare the MMIX for use in their laboratory’s workflow.

In high-throughput laboratories, which during the COVID-19 pandemic processed thousands of NAATs daily, automation of laboratory workflows was vital to achieve targets for turnaround times, capacity, and standardization. The development of laboratory automation for PCR testing focused on the sample preparation, purification, and the dispensing protocols for combining purified nucleic acid and PCR MMIX in microplate formats for PCR reactions [4]. Even data analysis was largely automated by software solutions [5]. Due to the difficulties in creating liquid classes for pipetting small amounts of viscous and often soapy reagents, as well as the need to determine the correct reagent volumes for varying test numbers, PCR MMIX preparation was frequently validated for routine use as a manual-only process. This occurred despite its very high value and critical importance to ensure quality [6], [7]. In regulated environments, it is important that checks are put in place to ensure that data integrity is preserved [8], but there are challenges with quality control procedures for PCR MMIX preparation. Options include gravimetric checks (which cannot distinguish between two reagents diluted in the same solvent), testing a sample of the batch (costing both time and reagents) or spectroscopic (requiring a spectrophotometer) [9].

**Fig. 1:**
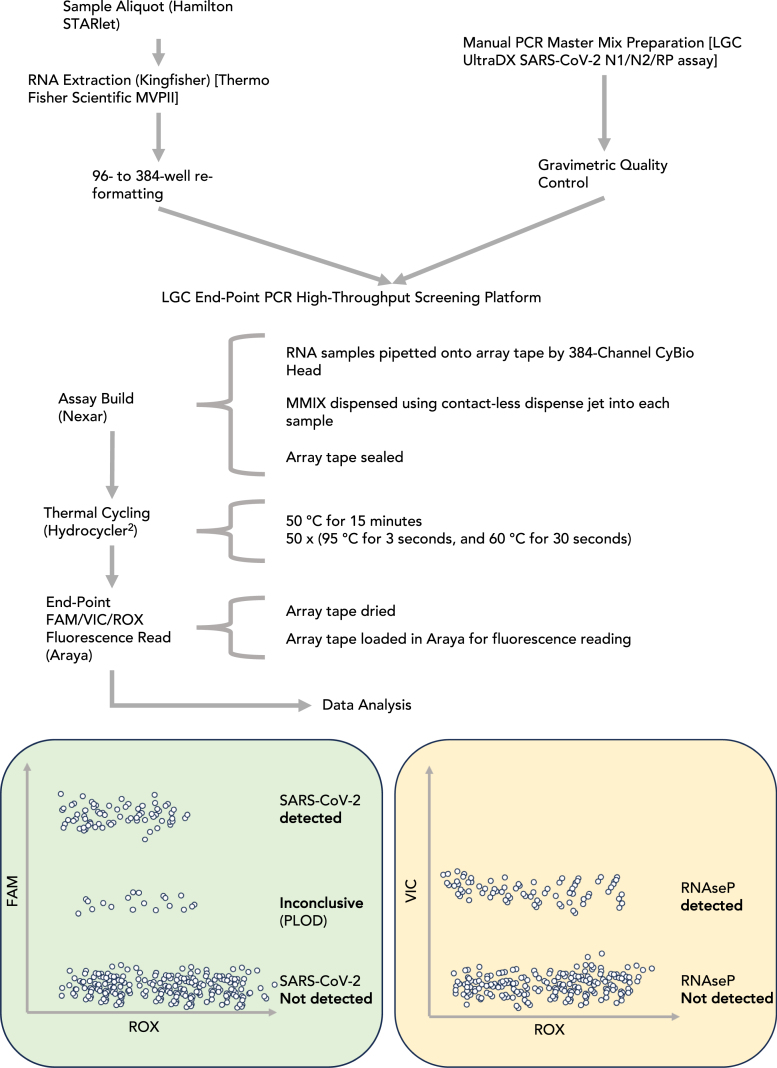
Process flow schematic for SARS-CoV-2 endpoint PCR (ePCR) workflow. Samples were purified using the Thermo Fisher Scientific MagMAX Viral Pathogen II kit and re-formatted from 96-deep-well to 384-well ANSI/SLAS microplates to prepare for assay build-in the Nexar Liquid handler 384-well Array Tape (AT). In parallel, PCR Master Mix (MMIX) was prepared and underwent a gravimetric quality control step before release for a run on the Nexar liquid handler. In practice, a run on the LGC ePCR High-throughput screening platform consisted of several (up to 16) 384-well microplates and a batch of PCR MMIX sufficient for all PCR reactions. The Nexar liquid handler first transfers samples with a 384-tip Dispense Pipet (DP) from the 384-well microplates to the AT before adding MMIX to each well of the tape with a Dispense Jet (DJ). The AT was then sealed, and reverse transcription and thermal cycling were conducted in a Hydrocycler^2^ (HC2) automated water bath. After the PCR reaction was completed, the Araya read the fluorescence of each well of the AT. Collected FAM (SARS-CoV-2 N1 and N2 targets) and VIC (human RNase P target) fluorescence data were normalized to ROX loading control and formed distinct clusters when plotted. The upper cluster in the FAM/ROX plot (green) was scored as SARS-CoV-2 detected, and occurs when both N1 and N2 targets amplify. The middle cluster, scored as Inconclusive or “Positive at Limit of Detection (PLOD),” refer to main text for further detail. The lower cluster was scored as SARS-CoV-2 Not Detected, corresponding with neither N1 nor N2 amplifying. The VIC/ROX plot (yellow) clusters into only two groups, scored as RNase P Detected, or RNase P Not Detected. (For interpretation of the references to color in this figure legend, the reader is referred to the web version of this article.)

An endpoint RT-PCR (ePCR) workflow was employed at large scale during the COVID-19 pandemic in the United Kingdom firstly at UK Biocentre, Milton Keynes and then at the Rosalind Franklin Laboratory, Leamington Spa (RFL) (Fig. 1) [10]. ePCR workflows, which have been proposed by numerous groups as a method of scaling up PCR testing operations [11], [12], [13], differ from the more commonly employed RT-quantitative PCR (RT-qPCR) workflows in the analysis stage, where in ePCR there is a single fluorescence read at the end of the PCR reaction, as opposed to once every thermal cycle in qPCR. The process was validated for manual PCR MMIX preparation, with a gravimetric quality control procedure [9]. In operation, meeting performance targets required creating large batches of PCR MMIX based on estimated test numbers. This approach led to a system susceptible to wastage and delays due to either overestimation or staff errors. Furthermore, the gravimetric quality control procedures were both slow and insufficient to differentiate between two of the reagents that were both diluted in Tris–HCl buffer.

At RFL we undertook a process automation project to fulfill the parallel requirements of maintaining consistently high-quality results while minimizing avoidable costs to the public purse due to wastage of valuable PCR reagents. The aim of this work was to develop an automated solution for MMIX preparation that would be equivalent to a trained human operator in terms of quality, but with greater consistency owing to the implementation of robotics.

At the time of writing, the World Health Organization has declared that COVID-19 is no longer a public health emergency [14]. In the UK, the screening of population samples for SARS-CoV-2 has been largely suspended [15]. While the risk of emergence of a novel immune-escape SARS-CoV-2 variant still exists, the application of high-throughput PCR screening is likely to be limited until the emergence of ’Pathogen X’ [16]. Nevertheless, automated PCR MMIX preparation methods have broad applications across molecular biology, and particularly in next-generation sequencing (NGS) library preparation applications. Such NGS applications are multi-stage workflows that often require samples to be treated with costly enzymatic methods that include adapter ligation, adenylation, and amplification [17].

## 2 Materials and methods

### 2.1 RNA purification

Viral RNA was extracted from control materials and patient swabs following the MagMAX Viral/Pathogen II (MVP II) nucleic acid isolation kit IFU on the Kingfisher Flex platform (Thermo Fisher Scientific, Waltham, Massachusetts, United States of America) (Fig. 1). Note that the extraction protocol concentrates the extracted RNA material: This is because input volume of patient sample is 200 μL, whereas elution volume is 60 μL. Therefore, assuming 100% efficiency of extraction, material at 1500 copies mL^−1^ pre-extraction, would become 5000 copies mL^−1^ post extraction. This concentration of material becomes relevant when calculating the limit of detection of the assay using different types of control materials (i.e., synthetic RNA, synthetic encapsulated RNA, or virus).

### 2.2 Master mix dispensing and PCR conditions

SARS-CoV-2 diagnostic NAATs were performed according to the LGC Biosearch Technologies (LGC, Teddington, United Kingdom) ePCR High-throughput screening platform IFU (Fig. 1), using manufacturer-supplied equipment (LGC’s Nexar, Hydrocycler^2^ (HC2) and Araya) and LGC’s SARS-CoV-2 ultra-high-throughput ePCR test reagents ( Table 1). Together, this system uses an ePCR method where fluorescence values of hydrolyzed target and control probes are taken at the end of the PCR reaction, unlike qPCR, [11], [12], [13].

Briefly, the Nexar is a modular in-line liquid handling system capable of offering highly scalable throughput owing to its Array Tape (AT) solution, which is a continuous polymer strip that is serially embossed with reaction wells in 384-well formats. A more detailed description of the validated ePCR system is as follows:

**Table 1:** LGC Biosearch Technologies SARS-CoV-2 ultra-high-throughput ePCR test reagent list and volumes per reaction.

Reagent	Target volume per reaction (μL)
RapiDXFire qPCR 5X Master Mix GF **(RDX)**	1
EpiScript RNase H- Reverse transcriptase **(EpiScript)**	0.125
UltraDX SARS-CoV-2 N1/N2/RP Assay **(OLIGO)**	0.05
SuperROX, reference dye **(ROX)**	0.025
Total Volume	1.2

A CyBio 384-pipet head (Dispense Pipet, DP) first transfers the purified RNA (3.8 μL) from a 384-well storage plate into the AT. A Dispense Jet 2.0 (DJ, an 8-tip, single jet solenoid micro-valve system, manufactured by LGC) aspirates approximately 520 μL of MMIX from a 96-deep-well Nexar Assay Plate (NAP) and then dispenses 1.2 μL of MMIX solution on top of the transferred RNA in the AT. In the deployed configuration, one DJ tip is used per array. The AT is sealed with an optically clear seal and transferred to the HC2 for the PCR reaction. The HC2 is an automated water bath system consisting of 3 separate tanks set at different temperatures (50 °C, 95 °C, and 60 °C). After the initial reverse transcriptase step (50 °C for 15 min), the AT is cycled 50 times between the 95 °C (3 s) and 60 °C (30 s) tanks, giving the denaturation, annealing and extension steps. At the end of the PCR reaction, the AT is manually dried by wiping with a lint free cloth and fed through the Araya, a single optic photomultiplier tube (PMT) reader equipped with 3 band pass filters matching the specification of dyes used in the reaction 2.3. A single measurement is taken for each well per wavelength and the raw data are corrected using the LGC’s proprietary deconvolution matrix to correct channel crosstalk. The processed data are exported as a .csv file for analysis.

### 2.3 PCR targets and result output

LGC’s UltraDX SARS-CoV-2 N1/N2/RP assay targets the SARS-CoV-2 nucleocapsid (N) gene. The assay uses the TaqMan PCR reaction principle [18] and uses a set of primers designed to anneal to two sections of the N gene [19]. Fluorescent TaqMan probes, consisting of a dye and a quencher, anneal downstream of the primers. Both N gene probes (referred to as N1 and N2) are labeled with a common fluorophore dye (FAM) with Black Hole Quencher (BHQ) and are pooled and detected with the same Araya channel. A third set of primers is designed to anneal to the human RNase P (RP) transcript and is used as an internal control. The respective probe is labeled with a VIC fluorophore dye with BHQ. ROX dye is added to the reaction both to provide an assay control that can be used to normalize the fluorescence signal and as a quality control step to ensure correct dispensing and addition of the Master Mix. FAM and VIC Probe Relative Fluorescence Units (RFU) was normalized to ROX RFU, i.e., normalized FAM (n_FAM) = FAM RFU/ROX RFU, and normalized VIC (n_VIC) = VIC RFU/ROX RFU. The use of a passive reference dye, such as ROX is now a widely adopted methodology for normalizing fluorescence values across the wells of a PCR reaction plate, with early examples of its use in the ABI qPCR instruments [20].

The resulting normalized values were used for calling of results:

• n_FAM ≥ 9 was “SARS-CoV-2 Detected”
• 4 ≤ n_FAM < 9 was “SARS-CoV-2 Inconclusive”, which was also termed Positive at Limit of Detection (PLOD)
• n_FAM < 4 was “SARS-CoV-2 Not Detected”
• n_VIC ≥ 1 was “RNase P Detected”
• n_VIC < 1 was “RNase P Not Detected”

Positive at Limit of Detection (PLOD) is defined as an ePCR test with normalized FAM fluorescence between 4 and 9. A PLOD outcome was regarded as a diagnostically inconclusive result as it was not consistent with the amplification of both N gene targets, nor was it consistent with no amplification. A re-test would also have had a high likelihood of giving an inconsistent result with the original test. Quality control for release of results of diagnostic tests requires a high degree of certainty over the outcome. Thus, if there were uncertainty over an outcome (as in the case with a PLOD result), the safest course of action was to re-test the patient and for them to continue their self-isolation until a “SARS-CoV-2 Not Detected” result was confirmed. PLOD results were hypothesized to occur due to the amplification of only one of the N1 or N2 targets, though this was never experimentally confirmed by us nor the vendor (LGC *personal communications*). Together, the ROX, SARS-CoV-2 and RNase P results were used by clinically trained personnel to approve a final result for release to NPEx (National Pathology Exchange - a national service for sending pathology test results and test requests, now known as Labgnostic).

### 2.4 Liquid handlers

This study compared two liquid handler platforms to manual MMIX preparation, the SPT Labtech (Melbourn, Hertfordshire, United Kingdom) Dragonfly Discovery (DF) and the Hamilton (Bonaduz, Switzerland) STARlet (SL).

Methods for creating and dispensing MMIX in NAP were developed for both DF and SL (DFD_MMIX.dfx and mmix_auto_v0.3.pkg respectively) and are available for download from https://github.com/Donald-OMIX/Liquid_Handler_Methods. A Hamilton STARlet fitted with a 96-channel pipetting head was used to re-format 96-well plates to 384-format where required.

### 2.5 Control material

For pilot experiments, control materials were supplied by Twist Bioscience (San Francisco, California, United States of America). The following synthetic RNA controls were used:

• Control 17, P.1, Gamma Variant (10^9^ copies mL^−1^)

For validation experiments and in routine use, Randox Laboratories Ltd (Crumlin, County Antrim, Northern Ireland, United Kingdom) supplied Qnostics, and Seracare (part of the LGC group) supplied AccuPlex control materials.

• Qnostics SARS-CoV-2 Q Control (SCV2QC) (500 copies mL^−1^)
• Qnostics SARS-CoV-2 Negative Q Control (TMNQC)
• AccuPlex SARS-CoV-2 Custom Control (1500 copies mL^−1^)
• AccuPlex SARS-CoV-2 Control (100,000 copies mL^−1^)

### 2.6 Pilot experiment protocol

Prior to designing a formal validation protocol, we conducted pilot experiments to optimize and determine the parameters of the method such that the automated and manual methods would give equivalent results in terms of both analytical and diagnostic sensitivity during validation. The protocol for the pilot experiments is as below:

Twist Gamma variant at 10^9^ copies mL^−1^ was diluted to 5000, 1500, and 500 copies mL^−1^ in elution buffer (EB) from the MVP II kit. Each dilution was then manually dispensed into separate 96-deep-well Kingfisher Plates (DWP). The three DWPs were re-formatted alongside an EB only DWP to a 384-format plate. It is worth noting that these concentrations are representative of post-extraction concentrations as opposed to the concentrations of crude samples. The 384-well plate was tested twice, firstly with manually-prepared MMIX, and secondly with the automated MMIX to allow for direct comparison. For each experiment, MMIX was manually-prepared first followed by the automated method. Owing to sharing equipment and space with a high-throughput COVID-testing operation, instrument availability was prioritized for patient screening over development work. The result of this prioritization was that within experiments, the Nexars and HC2s were kept consistent, but between experiments they differed.

Each array was analyzed according to SOPs and their standard acceptance criteria. ROX criteria for validity of results is as follows (for each 384-well array):

• 1600 < ROX mean relative fluorescence units (RFU) < 4800
• ROX coefficient of variation (CV) % ≤ 20

Dyes were normalized to the ROX RFU, i.e., normalized FAM (n_FAM) = FAM RFU/ROX RFU, and normalized VIC (nVIC) = VIC RFU/ROX RFU. The resulting normalized values were used for clinical calling whereby, n_FAM ≥ 9 is “Detected,” 4 ≤ n_FAM < 9 is “Inconclusive,” and n_FAM < 4 is “Not Detected.” Note that as the positive material was a synthetic COVID RNA construct, no RNase P signal was detected.

### 2.7 Validation experiment protocol

The validation protocol was organized around three major experiments:

1. ROX distribution and overall performance of the method, as well as cleanliness of the liquid handler. The objectives of this experiment were to confirm that the equipment:
  • Transferred correct volumes of reagent from the source vessels to the destination plate, by comparing ROX distribution from automated MMIX across different arrays.
  • Ran without error.
  • Was free from contamination (RNA/DNA) that might interfere with the subsequent validation processes.
  No COVID-positive material was run, only fresh EB, therefore any contamination due to the presence of SARS-CoV-2 RNA/DNA would be revealed after amplification.
2. ePCR sensitivity. The objective of this experiment was to determine if the automated workflows gave equivalent performance to that of the previously validated manual process through direct comparison with common MMIX reagents and reference materials. A small study was performed using synthetic SARS-CoV-2 material (Accuplex 100k copies mL^−1^) at 8 different concentrations (ranging from 2526 to 0 copies mL^−1^ to determine analytical sensitivity. The analytical sensitivity represents the smallest amount of substance in a sample that can accurately be measured by an assay and is also referred to as the lower limit of detection (LLoD). It is the lowest concentration of target in a specimen that can be consistently detected at an arbitrarily chosen rate (in this case 95%, LLoD95, see the European Pharmacopeia [21]), which requires the determination of the minimum number of target sequences per volume sample that can be detected in 95 percent of test runs. The LLoD95 was calculated using logit analysis (logistic regression). Acceptance criteria for LLoD95 was ≤ 1000 copies mL^−1^, taken from the Target Product Profile for SARS-CoV-2 diagnostics [22].
3. Concordance. A concordance study between manual and automated MMIX preparation was performed using patient samples to determine diagnostic sensitivity and specificity. A Cohen’s Kappa analysis compared the alignment of positive and negative diagnostic calls. Acceptance criteria: essential that the PPA, NPA, and OPA are ≥ 95%. Rates < 95% would require investigation and root cause analysis. In addition, the Cohen’s Kappa should report a value greater than 0.95.

### 2.8 Statistical analysis and data presentation

Positive percentage agreement (PPA), negative percentage agreement (NPA), overall percentage agreement (OPA), and Cohen’s Kappa were calculated according to FDA guidelines for evaluating diagnostic tests [23] when comparing a new test (automated MMIX) to a non-reference standard (manual MMIX on patient sample). Briefly, the PPA is considered here as the proportion of Positive (“Detected”) samples identified by manually-prepared MMIX that are also Positive in the automated-prepared MMIX. NPA is similar in concept, although it calculates the proportion of Negative (“Not Detected”) samples in the non-reference standards that are negative in the new test. OPA is the percentage of total subjects where both tests agree. Due to the intrinsically uncertain nature of “Inconclusive” results, they were not included in the calculation of OPA when identified as “Inconclusive” by the manually-prepared MMIX. However, if a result was definitively “Detected” or “Not Detected” in the manually-prepared MMIX, but was inconclusive in the automated method, these were included in the calculations. The net effect of doing this was to ensure that the automated MMIX method would only be approved for use should it give results that were equivalent to, or better than, the manual method.

A similar approach that excluded “Inconclusive” results as determined by the manual method was adopted for Cohen’s Kappa: calculated here as $k=\frac{P_{0}-P_{e}}{1-P_{e}}$ where P_0_ refers to the observed rate of overall agreement ($\frac{OPA}{100}$), and P${}_{e}$ is the hypothetical probability of random agreement.

P${}_{e}$ was estimated as: the “Detected” scoring rate of the manual method ($D_{m}$), multiplied by the “Detected” ($D_{a}$) scoring rate of the automated method; added to the “Not Detected” ($N_{m}$) scoring rate of the manual method, multiplied by the “Not Detected” ($N_{a}$) scoring rate of the automated method. Where the scoring rate is determined by the number of “Detected” or “Not Detected” results divided by the total number of results for the respective method. That is, $P_{e}=D_{m}\cdot D_{a}+N_{m}\cdot N_{a}$ [24].

As described above, this assay is a qualitative test (ePCR normalized FAM values for fully positive samples do not correlate to concentration of target DNA). Possible test results are “Positive”, “Negative”, and “Inconclusive”. For the purposes of analytical sensitivity testing, “Inconclusive” samples were classified as negative, producing a binary outcome. Depending on assay sensitivity, a sample of any given concentration has a probability (p) between 0 and 1 of being positive and a of 1-p of being negative. This can be estimated by fitting a logit model [25] to a dilution series spanning the concentrations where 0 < p < 1.

Data processing was carried out using R version 4.2.3 [26] with the following libraries:

• ggplot2 version 3.4.3
• flexible version 0.9.1
• dplyr version 1.1.2
• gridExtra version 2.3
• ggpubr version 0.6.0
• psych version 2.3.6
• caret version 6.0–94

Figures were produced with a mixture of Microsoft 365 version 16.83, and ggplot2 as above. Code is available on request.

## 3 Results

### 3.1 Qualitative evaluation of liquid handler platforms

We began by comparing the liquid handlers that we had in our possession. There were two clear front-runners for this project, the SPT LabTech Dragonfly Discovery (DF); and the Hamilton STARlet (SL) (Table 2). On the basis of its perceived ease-of-use, relatively low complexity, positive displacement pipetting performance, and dispense speed; we opted in the first instance to develop the DF platform for automated MMIX preparation.

**Table 2:** Qualitative comparison of MMIX preparation platforms.

Criteria	Manual	Dragonfly Discovery	Hamilton STARlet
Pipetting Technology	Air displacement hand-held pipets	Positive displacement	Air displacement
Liquid Level Detection	No	No	Yes – capacitance and pressure
Traceability	No – handwritten records only	No – handwritten records only	Yes – One-dimensional barcode tracking
Footprint	Medium	Small	Large and requires automation lab bench
Quality Control	Yes - gravimetric	Yes - spectral QC	Not necessary due to barcode tracking
Pipet Performance Checks	Daily gravimetric calibration	Not required	Automated daily and weekly maintenance
Time to Dispense 16 arrays in mins	105	4	15
Ad hoc Master Mix Preparation	No	Yes	Yes
Programming Complexity	None	Low complexity	High complexity

### 3.2 Overview of the Dragonfly discovery method for preparation and dispense of MMIX

We developed a method for the preparation and dispense of the MMIX using the SPT LabTech Dragonfly Discovery instrument that used 5 reservoirs and syringes, and a NAP. The system aspirated all the required reagents at once, but dispensed them in individual wells, thus effectively creating different MMIX in each well. The reagents were dispensed in order according to the SOP, i.e., RDX, ROX, OLIGO, and EpiScript (refer to Table 1 for reagent list). This specific order was enforced by modification of the dispense pattern to “typewriter”, as opposed to “serpentine”, and the aspiration location of the reagents. A “typewriter” dispense pattern refers to a fixed direction of dispense whereby, for each row or column, sequential wells are pipetted into from left to right or top to bottom, respectively. In contrast, a “serpentine” dispense pattern refers to a continuous dispensing pattern whereby the direction of dispense sequence, left–right and right-left; or top-bottom and bottom-top, alternates between sequential rows, or columns respectively. The serpentine dispense pattern tends to take a shorter amount of time to fill ANSI/SLAS microplates as fewer mechanical movements are required per row or column respectively.

We created two separate dispense layers: The first dispensed RDX, ROX and OLIGO, while the second dispensed only EpiScript. Our rationale for dividing the dispense into two stages was that EpiScript needed to be kept cold (−20 °C) until its addition to the mix, thus instead of aliquoting all the reagents into the reservoir at the beginning of the run, the operator dispensed EpiScript only after the first layer had been completed. After the first layer has completed, the machine was programmed to stop and ask for manual intervention. At that point, the operator added EpiScript in the dedicated reservoir and resumed the run. The two layers could be run using the “Sequence Launcher” function of the DF software. For a 16-array run, the DF took approximately 4 min from start to finish.

• Positions 7 and 8 of the reservoir tray were reserved for two standard reservoirs filled with up to 4.2 mL of RDX each to account for dead volume (syringe maximum capacity is 4 mL).
• Position 6 was dedicated to ROX, with 230 μL dispensed into a low-dead-volume (LDV) reservoir.
• Position 5 held OLIGO, with up to 440 μL in an LDV reservoir.
• Position 4 was reserved for EpiScript, up to 1.1 mL in an LDV reservoir.

We configured syringe channel 4 for minimum speed of aspiration and dispense of EpiScript, which is highly viscous. Slowing down aspiration and dispense speed is a liquid handling strategy for improving the accuracy of pipetting viscous liquids as high speeds can result in cavitation and the formation of droplets rather than a steady stream of liquid. After dispense, the MMIX plate was sealed and shaken for 30 s at 900 rpm on an Eppendorf Thermomixer (Hamburg, Germany) to ensure resuspension and mixing of reagents.

### 3.3 ROX RFU distribution in Dragonfly-prepared MMIX

**Fig. 2:**
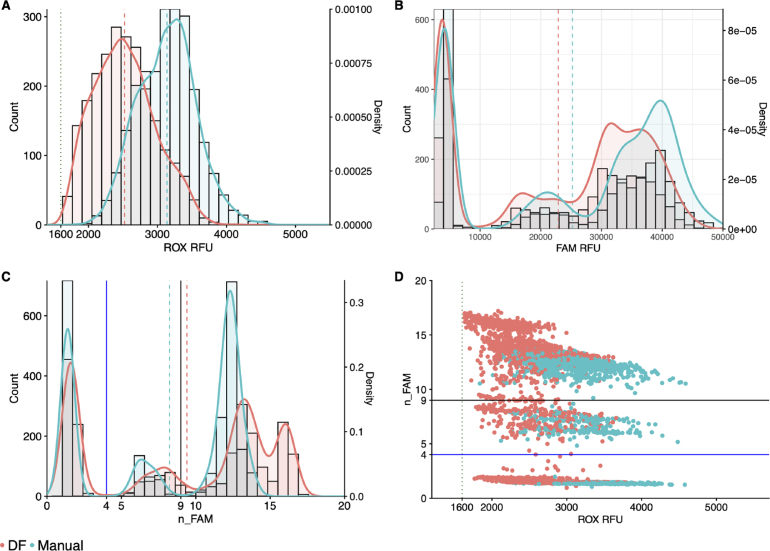
DF-prepared MMIX compared with manually-prepared MMIX endpoint RFU data from PCR reactions using dilutions of synthetic RNA positive control material (Twist) at and around the limit of detection. (A) ROX RFU kernel distribution and RFU counts. Dotted line represents the 1600 RFU cutoff. Dashed lines represent the arithmetic mean of ROX RFU. (B) Kernel Distribution and count for FAM RFU values. Dashed lines represent the arithmetic mean of FAM RFU. (C) Kernel Distribution and count for n_FAM values. Solid lines represent the “Inconclusive” PLOD (Blue) and “Detected” (Black) n_FAM cutoff for clinical calling. Dashed lines represent the arithmetic mean of n_FAM (D) Scatterplot n_FAM vs. ROX RFU. Data are pooled from six 384-well arrays for both conditions. Solid lines represent the “Inconclusive” PLOD (Blue) and “Detected” (Black) n_FAM cutoff for clinical calling. Dotted line represents the 1600 ROX RFU cutoff. Data are pooled from six 384-well arrays for both conditions. (For interpretation of the references to color in this figure legend, the reader is referred to the web version of this article.)

Using the above DF configuration 3.2, automated MMIX ROX statistics were within acceptance criteria (1600 < ROX mean relative fluorescence units (RFU) < 4800 and ROX coefficient of variation (CV) % ≤ 20 as detailed in 2.6) (Table 3). However, the distributions of ROX RFU and FAM RFU were both left-shifted in the DF-prepared MMIX compared to Manual (Fig. 2A & B). Lower ROX values could lead to a discrepancy in diagnostic calling as low ROX RFU will push n_FAM values higher (Fig. 2C & D).

**Table 3:** ROX statistics of Dragonfly and manually-prepared MMIX. Mean and SD calculated for each individual array, N = 384.

MMIX preparation method	Tape ID	Mean	SD	CV (%)
Dragonfly	738732	2359.7	276.8	11.7
	738733	2060.1	282.9	13.7
	852899	3024.8	349.4	11.6
	852901	2532.3	362.6	14.3
	852903	2623.7	369	14.1
	852905	2540.6	362.4	14.3

Manual	738734	3252.7	325	10
	738735	2962.5	313.7	10.6
	852900	3057.1	423	13.8
	852902	2951.4	417.8	14.2
	852904	3200.1	394.4	12.3
	852906	3398.7	434.5	12.8

### 3.4 Addition of emulsifying agent (Tween-20) to ROX

A root cause analysis was performed to understand the reasons for the low ROX RFU values observed 3.3. Under dispensing was ruled out as a volumetric check revealed dispense accuracy and precision within our acceptance criteria (data not shown). We postulated that plastic binding might be responsible for the drop in RFU as rhodamine derivatives (such as the ROX fluorophore) adsorb to plastics [27]. We decided to overcome this potential issue by adding an emulsifying agent to ROX, Tween-20. This non-ionic surfactant was normally added to keep the ROX fluorophore in solution, however this agent was later removed by the manufacturer as it was deemed unnecessary for their final product formulation. We performed a preliminary experiment where 0.05% Tween-20 (provided by LGC) was added to a sub-aliquot of ROX, and the MMIX was then prepared both manually and with the DF, and the experiment described above 3.3 was repeated.

**Fig. 3:**
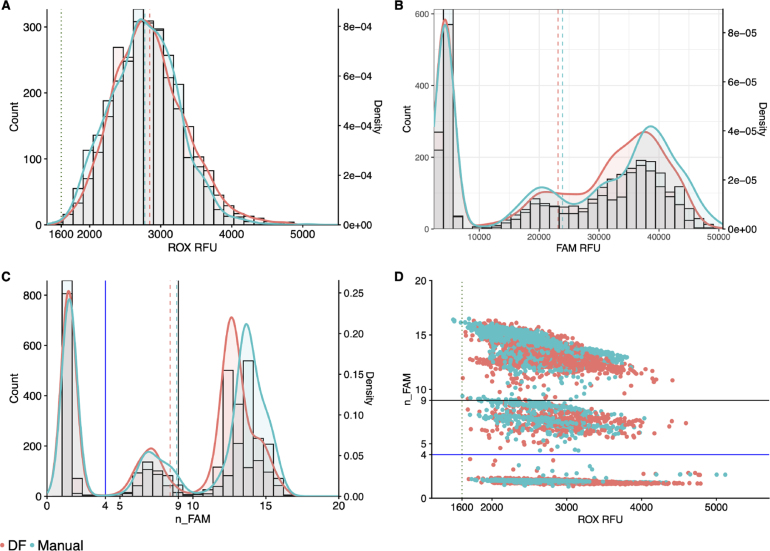
DF-prepared MMIX compared with manually-prepared MMIX after addition of 0.05% Tween-20. endpoint RFU data from PCR reactions using dilutions of synthetic RNA positive control material (Twist) at and around the limit of detection. (A) ROX RFU kernel distribution and RFU counts. Dotted line represents the 1600 ROX RFU cutoff. Dashed lines represent the arithmetic mean of ROX RFU. (B) Kernel Distribution and count for FAM RFU values. Dashed lines represent the arithmetic mean of FAM RFU. (C) Kernel Distribution and count for n_FAM values. Solid lines represent the “Inconclusive” PLOD (Blue) and “Detected” (Black) n_FAM cutoff for clinical calling. Dashed lines represent the arithmetic mean of n_FAM. (D) Scatterplot n_FAM vs. ROX RFU. Solid lines represent the “Inconclusive” PLOD (Blue) and “Detected” (Black) n_FAM cutoff for clinical calling. Dotted line represents the 1600 ROX RFU cutoff. Data are pooled from seven 384-well arrays for both conditions. (For interpretation of the references to color in this figure legend, the reader is referred to the web version of this article.)

The addition of Tween-20 brought ROX statistics and distribution for both the DF and manually-prepared MMIX into alignment (Table 4 & Fig. 3A). In addition, the FAM distributions between the manual and DF-prepared MMIX were also closely aligned (Fig. 3B). Correspondingly, they showed overlapping n_FAM distributions (3C & D). Array Tape ID 740959 resulted in a ROX CV % > 20. While this figure exceeded the acceptance criteria, the most likely cause of this issue is attributable to a Nexar mis-dispense rather than a DF mis-dispense. This attribution can be explained as follows: MMIX was prepared in one large batch, then aliquoted equally into multiple wells of a Nexar plate, which eliminated variation in MMIX preparation as a major source of variation between arrays. The DJ, which was an 8-channel contact-free liquid dispenser, aspirated MMIX from the Nexar plate, and was programmed to dispense one channel per array. Therefore, mis-dispense, or variable dispense of a DJ channel, would manifest as increased CV %. As CV % measures the relative dispersion of data points in a data series around the mean, it was routinely used as a metric to scrutinize and ensure the correct functioning of the Nexar at RFL. In addition, the DF aspirates all the required volume for ROX at once. If not enough liquid is present in the reservoir, air will be aspirated instead creating a gap between the plunger and the meniscus, which would only impact the final dispense of a run (Array Tape ID 740960), rather than the middle dispense.

In our experiments, the inclusion of Tween-20 did not yield any discernible impact on the detection of SARS-CoV-2 by the assay. Nevertheless, as the manufacturer had discontinued the addition of this emulsifier in their assay, re-introduction of Tween-20 to the reagent formulation would have required an assay re-validation to confirm that there were no negative performance impacts to the validated end-to-end SARS-CoV-2 screening assay. This activity was out of scope of the automation project, so we opted not to proceed with Tween-20 addition.

**Table 4:** ROX statistics of Dragonfly and manually-prepared MMIX with Tween-20 added. Mean and SD calculated for each individual array, N = 384.

MMIX preparation method	Tape ID	Mean	SD	CV (%)
Dragonfly	740958	3059.4	511.6	16.7
	740959	2760.4	586.8	21.3
	740960	2828.2	540.3	19.1
	807077	2699.7	504	18.7
	807078	3106.5	479.8	15.4
	807079	2672.7	310.8	11.6
	807080	2792.2	391.7	14

Manual	740967	2637.4	342.6	13
	740968	2569	391	15.2
	740969	2230.7	348.1	15.6
	807081	2796.5	369.8	13.2
	807082	3088	317.1	10.3
	807083	3158.4	317.6	10.1
	807084	2956.5	506.8	17.1

### 3.5 UV–VIS spectroscopy for quality control of prepared MMIX

At RFL, before usage in routine clinical analysis, MMIX was subjected to a gravimetric quality control check (QC). While this method is simple and does not require specialized equipment (besides a balance), it was not suitable for detecting a common operator mistake: the swapping of ROX and OLIGO blend volumes. As both reagents are diluted in the same buffer (Tris–HCl based buffer), their mass:volume ratio is nearly identical. Such an error would lead to substantial differences in the clinical calling, as the ratio between the normalizer (ROX) and the real signal (FAM/VIC probe blend) would be incorrect. To overcome this issue, we developed a new method whereby the absorbance of the MMIX is measured at defined wavelengths, namely 502, 550, and 585 nm, which are the peak absorbance wavelengths of FAM, VIC, and ROX, respectively. By calculating a ratio between the different peaks, it was possible to define a range within which an MMIX is deemed correct (3 SD from the mean).

We compared MMIX created either manually or with the DF and simulated common errors in MMIX preparation (e.g., swapping the volumes of ROX and OLIGO).

**Fig. 4:**
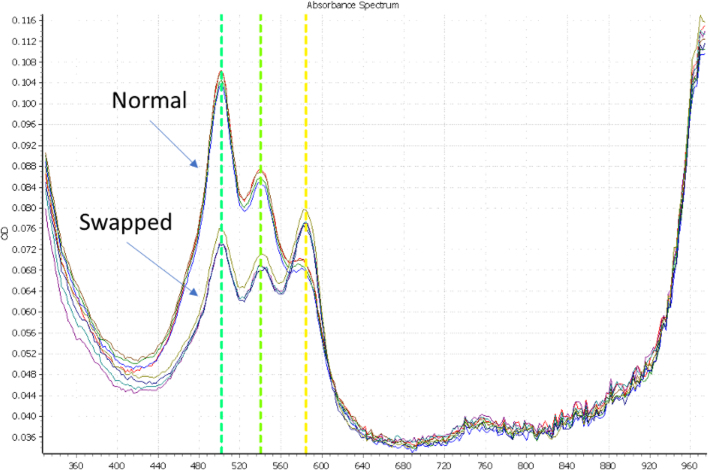
ABS scan, comparing the absorption spectra of “Normal” MMIX, and a “Swapped” MMIX, where the volumes of the ROX and OLIGO are exchanged. N = 4. Each line represents a separate replicate.

**Fig. 5:**
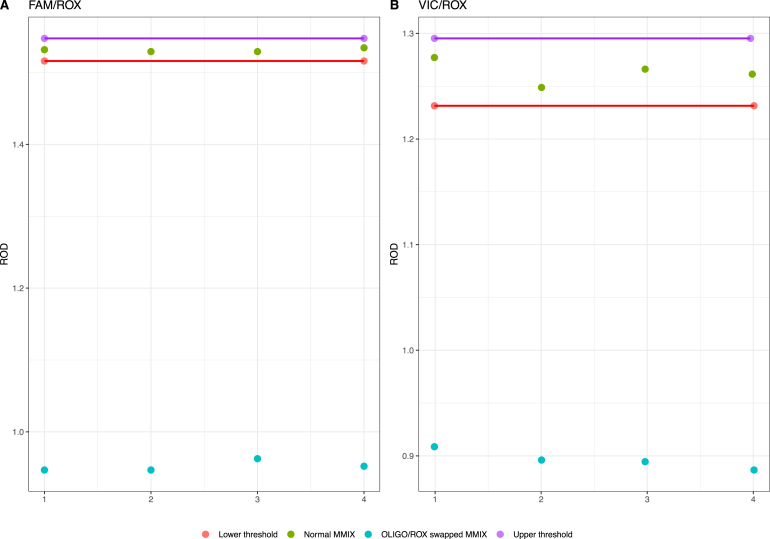
Ratio of FAM/ROX (A) and VIC/ROX (B), with upper and lower limits defined as ± 3 SD from the mean. N = 4. ROD = Relative Optical Density.

For this experiment, we used a PheraSTAR plate reader on loan from BMG LABTECH (Ortenberg, Germany). 10 μL of MMIX (both correct and swapped) were dispensed into 4 wells of a flat bottom plate (Corning UV micro-star LV, Corning, New York, United States of America) and a spectral scan was performed. As depicted in Fig. 4, the absorption spectral scan (ABS) revealed clearly defined peaks at the predicted wavelengths for the normal and swapped MMIX. By calculating the ratio between FAM/ROX and VIC/ROX, we observed a clear cluster separation between the two MMIXs (Fig. 5A & B).

**Fig. 6:**
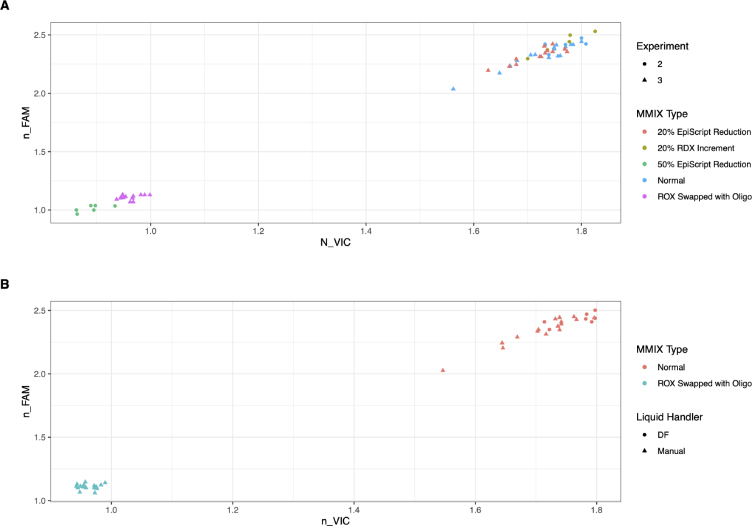
Clustering of normalized FAM and normalized VIC Absorption spectra showing simulated errors. A) Simulated Common Errors. Variation in the MMIX composition exhibited a defined OD signature when an MMIX sample was read through the plate reader. Common operator errors were replicated. Swapping ROX with OLIGO and addition of 50% less EpiScript resulted in defined clusters separated from the correctly-prepared MMIX. Other errors such as a reduction of 20% to RDX volume or a 20% reduction of EpiScript, while less common but nonetheless plausible, did not show good cluster separation from the correctly-prepared MMIX. Data were pooled from two separate experiments (2 and 3). (B) Comparison of DF-prepared MMIX, manually-prepared MMIX, and with ROX and OLIGO swapped. While Swapping ROX and OLIGO resulted in a clearly separated cluster, no clustering was observed between DF and manually-prepared MMIX supporting the conclusion that the DF performed similarly to an expert operator. Note, Tween-20 was added to ROX in all experiments.

To investigate further the sensitivity of the spectroscopic QC step, we tested several other possible MMIX errors (Fig. 6A). We also compared DF and manually-prepared MMIX and observed that the clusters overlapped (Fig. 6B), again confirming the ability of the DF to produce MMIX of a quality comparable to an experienced operator.

These data together suggest that the spectroscopic QC assay could be a highly sensitive method for detecting incorrectly prepared MMIX, particularly for cases that could not be otherwise detected gravimetrically. Similarly, other “errors” like a 50% reduction in volume of EpiScript can also be detected. However, despite the sensitivity of the instrument, small variations in the EpiScript volume (20% reduction) could not be detected spectroscopically using our method. There is the potential for use of gravimetric detection, as a variation of 20% will shift the weight of MMIX by 2.1%, raising the possibility of using both QC methods in concert as a more robust detection system, should the application require it.

Despite its equivalent performance to a skilled manual operator, we were unable to take the DF to a routine operational environment as to do so would have required the spectroscopic QC, and, unfortunately, the PheraSTAR plate reader loan unit had to be returned. We therefore proceeded to develop our other liquid handler platform, the Hamilton STARlet. Pivoting to an alternate platform presented an opportunity to utilize the pipet log, liquid level detection and tracking features of the STARlet platform, which together could bypass the requirement for the QC step entirely.

### 3.6 Overview of the STARlet method for preparation and dispense of MMIX

We designed and programmed the STARlet method to maximize reagent recovery in the event of the method aborting due to (e.g.) an aliquot running out, or the operator loading labware or reagents incorrectly. To achieve this, we implemented liquid level detection and barcode tracking of all reagent containers. We developed the logic of the method to minimize the risk of wastage of expensive reagents while respecting the requirements for keeping the EpiScript cold (Algorithm 1). Briefly, RDX, ROX, and OLIGO were not mixed until the method confirmed that all three reagents were available in sufficient quantity. RDX is dispensed first as it is a soapy reagent added in bulk that if left in the tip while the other reagents were aspirated would begin to drip (perhaps due to the detergent in the RDX lowering the cohesive forces of the liquid). The method then requested EpiScript, and if sufficient quantity was confirmed, the method proceeded to combine the EpiScript with the mixed reagents.

**Figure:**
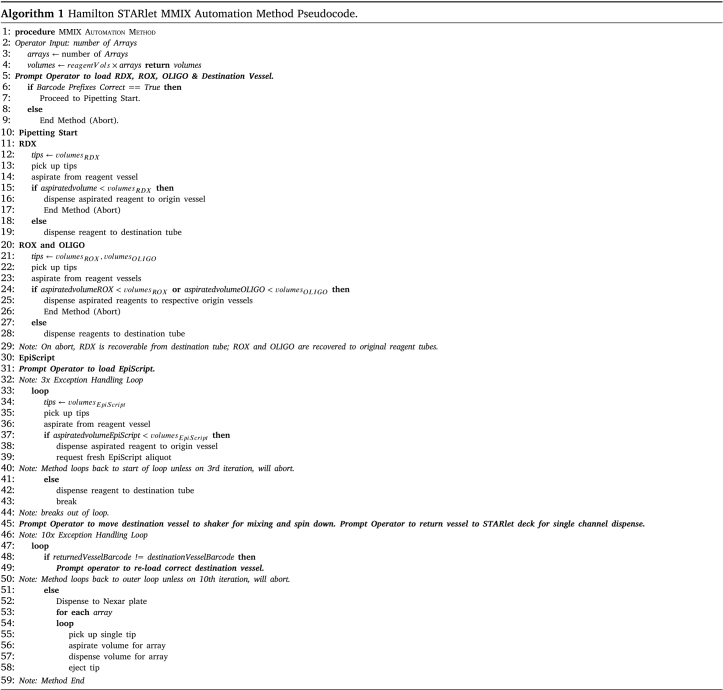

### 3.7 Hamilton STARlet pipetting performance compared to a manual operator

**Fig. 7:**
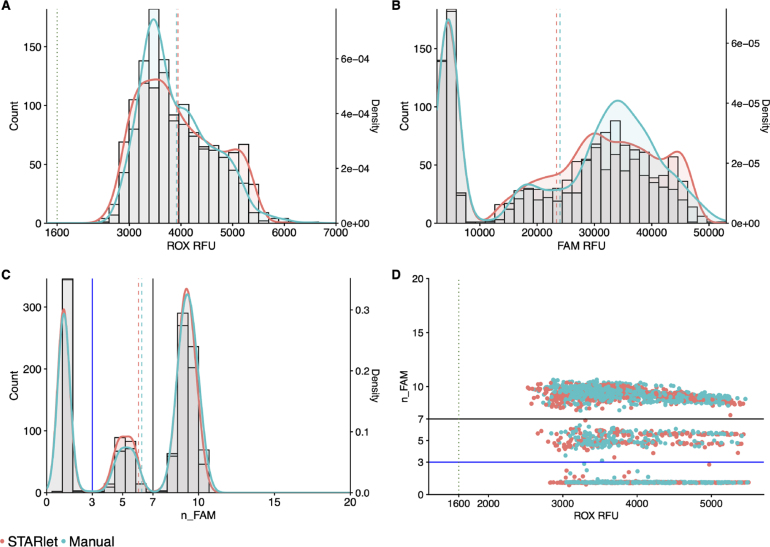
STARlet-prepared MMIX compared with manually-prepared MMIX endpoint RFU data from PCR reactions using dilutions of synthetic RNA positive control material (Twist) at and around the limit of detection. (A) ROX RFU kernel distribution and RFU counts. Dotted line represents the 1600 ROX RFU cutoff. Dashed lines represent the arithmetic mean of ROX RFU. (B) Kernel Distribution and count for FAM RFU values. Dashed lines represent the arithmetic mean of FAM RFU. (C) Kernel Distribution and count for n_FAM values. Solid lines represent the “Inconclusive” PLOD (Blue) and “Detected” (Black) n_FAM cutoff for clinical calling. Dashed lines represent the arithmetic mean of n_FAM. (D) Scatterplot n_FAM vs. ROX RFU. Solid lines represent the “Inconclusive” PLOD (Blue) and “Detected” (Black) n_FAM cutoff for clinical calling. Dotted line represents the 1600 RFU cutoff. Data are pooled from three 384-well arrays for both conditions. It is worth noting that in this experiment we observed a higher average ROX RFU for both the STARlet and manually-prepared MMIX. This was caused by a higher concentration of fluorophore in the manufacturer-supplied ROX reagent. However, the higher concentration of normalizing dye shifted the n_FAM values, thus the thresholds for just this pilot experiment were shifted accordingly to match the shift in the clusters. (For interpretation of the references to color in this figure legend, the reader is referred to the web version of this article.)

Comparison of manually-prepared MMIX with Hamilton STARlet (SL)-prepared MMIX showed a high degree of overlap for ROX, and FAM RFU distributions (Fig. 7). Furthermore, ROX statistics were within acceptance criteria (1600 < ROX mean relative fluorescence units (RFU) < 4800 and ROX coefficient of variation (CV) % ≤ 20 as detailed in 2.6) (Table 5). Based on the promise shown by these data, we validated the method for routine processing of clinical samples.

**Table 5:** ROX statistics of Hamilton STARlet and manually-prepared MMIX. Mean and SD calculated for each individual array, N = 384.

MMIX preparation method	Tape ID	Mean	SD	CV (%)
Hamilton	596217	4623.4	587.8	12.7
	596219	3913.9	510	13
	596221	3284	361.2	11

Manual	596218	4440.7	638.6	14.4
	596220	3787.1	597.1	15.8
	596222	3502.9	356.7	10.2

### 3.8 Validation of Hamilton STARlet automated PCR MMIX preparation for routine diagnostic use

The first validation experiment sought to assess the overall performance of the method through a formal assessment of ROX statistics (Table 6). Note that the first dispensed array (567099) showed higher ROX CV % (19%) compared to the other dispensed arrays. Given that the MMIX batch was the same for the first 4 arrays (567099–567102), we attributed this issue to reduced dispensing performance of the DJ tip on the Nexar, which was a known and recurring issue, described previously in 3.4. These data were nevertheless included in the results calculations as they still met the criteria for validity of results: 1600 < ROX mean relative fluorescence units (RFU) < 4800 and ROX coefficient of variation (CV) % ≤ 20 as detailed in 2.6.

**Fig. 8:**
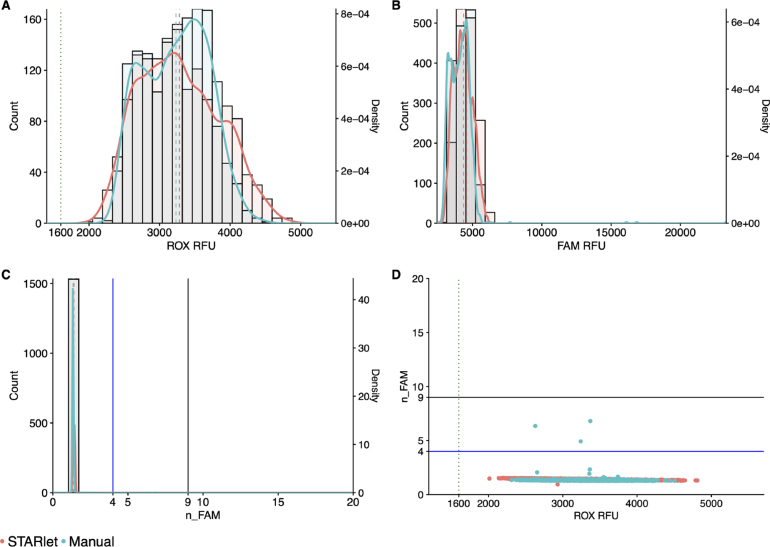
STARlet-prepared MMIX compared with manually-prepared MMIX ndpoint RFU data from PCR without inclusion of positive control material to check for contamination. (A) ROX RFU kernel distribution and RFU counts. Dotted line represents the 1600 ROX RFU cutoff. Dashed lines represent the arithmetic mean of ROX RFU. (B) Kernel Distribution and count for FAM RFU values. Dashed lines represent the arithmetic mean of FAM RFU. (C) Kernel Distribution and count for n_FAM values. Solid lines represent the “Inconclusive” PLOD (Blue) and “Detected” (Black) n_FAM cutoff for clinical calling. Dashed lines represent the arithmetic mean of n_FAM. (D) Scatterplot n_FAM vs. ROX RFU. Solid lines represent the “Inconclusive” PLOD (Blue) and “Detected” (Black) n_FAM cutoff for clinical calling. Dotted line represents the 1600 ROX RFU cutoff. Data are pooled from four 384-well arrays for both conditions. (For interpretation of the references to color in this figure legend, the reader is referred to the web version of this article.)

This experiment aimed to confirm that the Nexar system met the cleanliness criteria necessary for continuing the validation protocol, as well as confirming that the ROX distribution fell within the acceptable criteria. Only elution buffer was utilized as the sample, therefore the expected outcome was that all wells should yield negative results, with an accepted limit of less than 2 “Inconclusive” positive at limit of detection (PLOD) results per array. The distribution of ROX and FAM between the two MMIX preparation methods was highly comparable (Fig. 8A & B). The observation that there were no positive wells and a total of 3 inconclusive wells from across all the arrays (equal to 0.1% of total wells) confirmed that the instruments and methods were contaminant free over the 8 examined arrays (Fig. 8C & D).

**Fig. 9:**
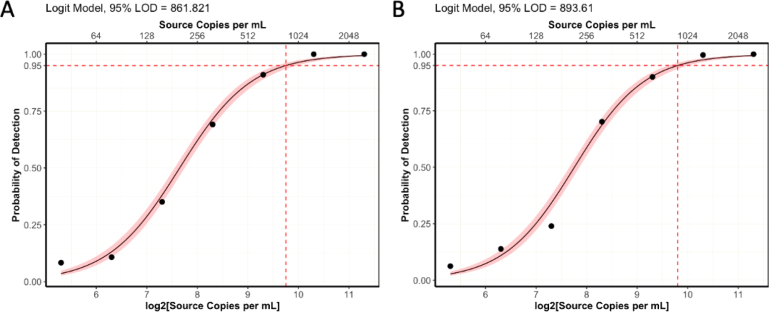
Logit models for analytical sensitivity of (A) Manual process (B) Automated process. N = 96 per concentration for both conditions. Pink shaded region represents 95% confidence interval. (For interpretation of the references to color in this figure legend, the reader is referred to the web version of this article.)

**Fig. 10:**
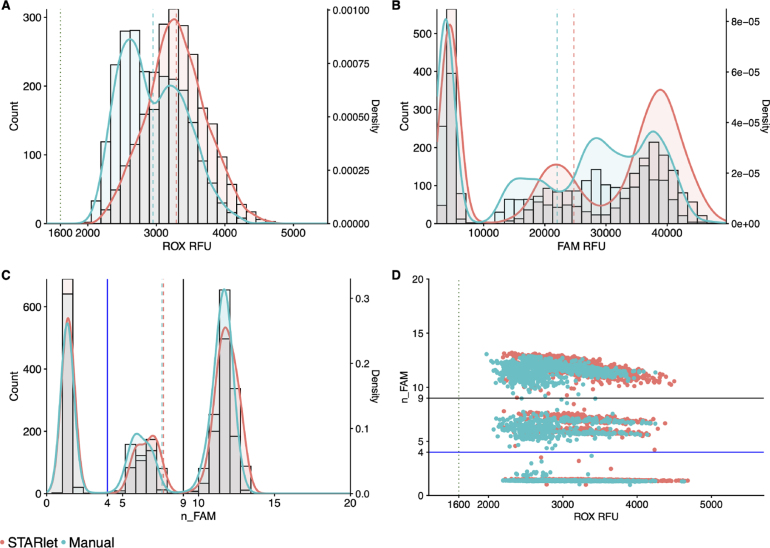
STARlet-prepared MMIX compared with manually-prepared MMIX endpoint RFU data from PCR reactions using dilutions of synthetic RNA positive control material (Seracare AccuPlex) at and around the limit of detection. (A) ROX RFU kernel distribution and RFU counts. Dotted line represents the 1600 ROX RFU cutoff. Dashed lines represent the arithmetic mean of ROX RFU. (B) Kernel Distribution and count for FAM RFU values. Dashed lines represent the arithmetic mean of FAM RFU. (C) Kernel Distribution and count for n_FAM values. Solid lines represent the “Inconclusive” PLOD (Blue) and “Detected” (Black) n_FAM cutoff for clinical calling. Dashed lines represent the arithmetic mean of n_FAM (D) Scatterplot n_FAM vs. ROX RFU. Solid lines represent the “Inconclusive” PLOD (Blue) and “Detected” (Black) n_FAM cutoff for clinical calling. Dotted line represents the 1600 ROX RFU cutoff. Data are pooled from six 384-well arrays for both conditions. (For interpretation of the references to color in this figure legend, the reader is referred to the web version of this article.)

The second validation experiment assessed the analytical sensitivity of the ePCR assay comparing MMIX prepared with the automated STARlet method and manual workflows. We prepared a dilution series of synthetic control material (AccuPlex SARS-CoV-2 100,000 copies mL^−1^) (n = 96 for each concentration). The dilution series was tested against both automated and manually-prepared MMIX. The experiment was repeated three times, and we performed a logit analysis (Fig. 9) to determine the analytical sensitivity (LLoD95) ( Table 7). The analytical sensitivity for both MMIX preparation methods met the acceptance criteria of ≤ 1000 copies mL^−1^, with 0 false positives. In addition, all 12 arrays passed the ROX volumetric dye acceptance criteria (CV% ≤ 20%) ( Table 8) and the overall distributions of ROX between automated and manually-prepared MMIX were highly comparable (Fig. 10A). Despite a slight right-ward shift in FAM distribution (Fig. 10B), n_FAM was also closely aligned (Fig. 10C & D).

**Table 6:** ROX statistics for formal validation experiment of STARlet method. Mean and SD calculated for each individual array, N = 384.

MMIX preparation method	Tape ID	Mean	SD	CV (%)
Hamilton	567099	3095.2	586.8	19
	567100	3008.2	438.3	14.6
	567101	3346.2	421.7	12.6
	567102	3673.8	517	14.1

Manual	567103	3106.6	396.8	12.8
	567104	3308.4	354.6	10.7
	567105	3656.5	301.9	8.3
	567106	2861.5	324.8	11.4

The final validation experiment sought to determine diagnostic sensitivity through comparison of the automated MMIX procedure with the validated manual procedure using true patient samples. A set of 12 384-well storage plates containing purified patient samples were chosen at random to be first subjected to the validated in-use manual MMIX process, and then by the automated MMIX process.

**Table 7:** Summary of analytical sensitivity of automated and manually-prepared MMIX.

Method of MMIX preparation	Replicate ID	LLoD95 (copies mL^−1^)
Hamilton	Titer 1	931
	Titer 2	808
	Titer 3	935

Manual	Titer 1	831
	Titer 2	837
	Titer 3	918

**Table 8:** ROX statistics for ePCR sensitivity experiments. Mean and SD calculated for each individual array, N = 384.

MMIX preparation method	Tape ID	Mean	SD	CV (%)
Hamilton	567110	3038	395.9	13
	567111	3247.4	377.1	11.6
	567112	3572.9	487.7	13.6
	567113	3342.5	419.6	12.6
	567114	3340.9	345.2	10.3
	567115	3195.9	381.3	11.9

Manual	567116	3274.5	449.3	13.7
	567117	3122.1	575.2	18.4
	567118	3009.5	344.4	11.4
	567119	2992.4	420.3	14
	567120	2743.9	221.7	8.1
	567121	2561.2	263.5	10.3

The performance of the ROX volumetric dye passed acceptance criteria for 23 out of 24 arrays. Tape ID 567232 had a cluster of 6 wells with ROX RFU > 5000 RFU. Root cause analysis revealed that this array was dispensed by Nexar DJ tip 5, which had previously recorded performance issues. All other wells had ROX RFU within the expected range, therefore excluding these 6 wells from the analysis allowed for the confirmation that the MMIX preparation by the STARlet and the MMIX dispense by the Nexar platform met requirements for diagnostic validity ( Table 9).

**Fig. 11:**
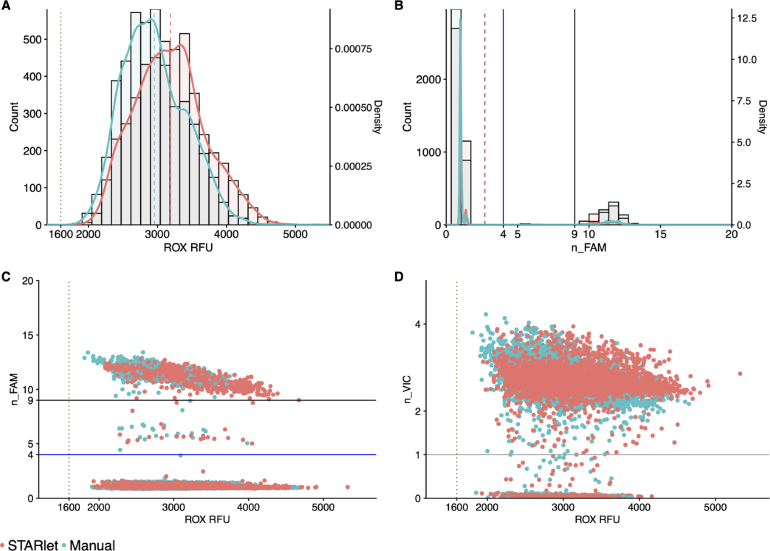
STARlet-prepared MMIX compared with manually-prepared MMIX endpoint RFU data from PCR reactions using patient samples and production control material. (A) ROX RFU kernel distribution and RFU counts. Dotted line represents the 1600 ROX RFU cutoff. Dashed lines represent the arithmetic mean of ROX RFU. (B) Kernel Distribution and count for n_FAM values. Solid lines represent the “Inconclusive” PLOD (Blue) and “Detected” (Black) n_FAM cutoff for clinical calling. Dashed lines represent the arithmetic mean of n_FAM (C) Scatterplot n_FAM vs. ROX RFU. Data are pooled from six 384-well arrays for both conditions. Solid lines represent the “Inconclusive” PLOD (Blue) and “Detected” (Black) n_FAM cutoff for clinical calling. Dotted line represents the 1600 ROX RFU cutoff. (D) Scatterplot n_VIC vs. ROX RFU. Solid lines represent the cutoff for clinical calling of n_VIC. Dotted line represents the 1600 ROX RFU cutoff. Data are pooled from twelve 384-well arrays for both conditions. (For interpretation of the references to color in this figure legend, the reader is referred to the web version of this article.)

ROX and n_FAM distribution were again very similar between automated and manually-prepared MMIX methods (Fig. 11A & B). Correlation between n_FAM vs. ROX and n_VIC vs. ROX also showed overlapping distributions between the two MMIXs (Fig. 11C & D). A Cohen’s Kappa analysis of the diagnostic results showed close correlation and agreement on results (≥ 95%) between the two MMIX preparation methods when testing patient samples ( Table 10 & Table 11).

Together, the data collected during the validation demonstrated that the automated MMIX method gave results equivalent to those of trained and experienced operators. The method was therefore approved for routine operational use in the laboratory.

**Table 9:** ROX statistics of all arrays from the concordance study. Mean and SD calculated for each individual array, N = 384.

MMIX preparation method	Tape ID	Mean	SD	CV (%)
Hamilton	567228	2683.8	405.9	15.1
	567229	3412.3	406.5	11.9
	567230	3203.6	452.4	14.1
	567231	3110.4	329.2	10.6
	567232	3372.4	1150.2	34.1
	567233	3159.5	328.3	10.4
	567234	3206.8	487.6	15.2
	567235	2652.5	307.1	11.6
	567265	3193.4	419.8	13.1
	567266	3608.3	456.6	12.7
	567267	3564.2	508	14.3
	567268	3090.8	591.9	19.2

Manual	567173	2949	385.6	13.1
	567174	2585.8	334.2	12.9
	567175	2625.3	329.8	12.6
	567176	3019.2	276.3	9.2
	567177	3003.5	487.6	16.2
	567178	3318.9	371.6	11.2
	567179	3375.1	410.2	12.2
	567180	3000.4	493.4	16.4
	819028	3277.2	392.3	12
	819059	2710.6	297.1	11
	819061	2930.1	225.5	7.7
	819078	2654.5	348.5	13.1

**Table 10:** Cohen’s Kappa analysis of percent agreement. PPA = positive percentage agreement, NPA = negative percentage agreement, OPA = overall percentage agreement. Cohen’s Kappa considers the level of agreement between the two MMIX preparation methods, but takes into account the probability of their agreement occurring by chance. The calculations are described more fully in 2.8.

Class	%	Cohen’s Kappa
PPA	98.71	0.95
NPA	98.52	
OPA	98.55

**Table 11:** Confusion matrix of concordance results.

		Manual		
		Detected	Not Detected	Inconclusive
Automated	Detected	689	2	4
	Not Detected	1	3524	44
	Inconclusive	8	51	249

## 4 Discussion

For development of an automated MMIX preparation method, we initially favored the SPT Labtech Dragonfly Discovery liquid handler. This hardware platform uses positive displacement pipetting technology where liquid directly contacts the piston. This is advantageous over air displacement, or liquid displacement, as there is no compressible air gap between liquid and piston to consider when transferring liquids [17]. Conversely, air displacement liquid handlers (such as the Hamilton STARlet) trap air between the piston and the sample liquid. The volume of the air cushion is affected by external factors such as temperature (ambient and sample) and slow liquid movement due to high viscosities, both of which might hinder aspiration and dispense volumes. Optimization of pipetting accuracy and precision can be achieved through a labor-intensive process of optimization of liquid class parameters including aspiration/dispense speed, swap speed, settling time, transport air volume, and proprietary technologies such as Hamilton’s pressure monitoring and anti-droplet control. Considering the high viscosity and low temperature of the reagents in the MMIX formulation, a positive displacement liquid handler will minimize pipetting errors with the least amount of optimization required [17]. Thus, with some minor adjustments to aspiration and dispense speed, we very quickly achieved accurate dispensing performance. However, as the Dragonfly Discovery lacked a barcode reader and liquid level detection technologies, it was not able to provide the level of process control we required for the operational environment. Key considerations in evaluating, developing, and operationalizing automated workflows are also the operator experience of its use, compliance requirements, and the need for process controls [28]. We therefore shifted development to the Hamilton STARlet platform, which could provide liquid level detection, a pipet log audit trail, a customized user experience, and a tracking database.

Our deployed method was highly customized to fit operational requirements and used both custom and built-in liquid classes. We implemented liquid level detection and barcode tracking of all reagents’ containers prior to the mixing of any reagents until the method confirmed that all reagents were available in sufficient quantity. If the method did not confirm there was sufficient quantity, it would recover all aspirated liquids by dispensing them back to their source containers. We designed the overall logic of the method to maximize the opportunity for reagent recovery in the event of the method aborting (Algorithm 1). While all applications differ, and this exact method will not be directly applicable to other workflows, the underlying logic of it is re-usable. It is an example of how it is possible to minimize the risk of reagent wastage while providing consistent results.

As the end-to-end assay was not completely novel, and with the novelty limited to only the MMIX preparation step, we could validate the automated method through a method transfer exercise rather than a *de novo* validation: a method transfer need only confirm that results from the new automated MMIX preparation method did not differ significantly from the existing validated manual method [29]. With our method transfer validation protocol, we showed that both the raw endpoint fluorescence data and the results calling generated by our automated Hamilton STARlet method were equivalent and highly concordant with data generated by the manual method. While small shifts in the distribution of raw endpoint fluorescence were observed comparing the Hamilton STARlet and the manual methods, these were not sufficient to affect the clinical calling of results as the clusters of the n_FAM still fell within the previously set thresholds of ≥ 9 for a “Detected” result and < 4 for a “Not Detected” result. Interestingly, with the Dragonfly Discovery dispense, we did note a right-ward shift of the normalized n_FAM data largely due to a left-ward shift in the ROX fluorescence data, which we presumed was due to ROX dye precipitating out of solution and binding to the plastic of the reagent troughs. The overall effect that would have on the clinical results calling, while keeping the same thresholds, would be for results in the “Inconclusive” category to start to shift into the “Detected” category, and likewise “Not Detected” results could foreseeably move into the “Inconclusive” category. As these results were used to decide on treatment options for patients, the avoidable potential for uncertainty and delays over results represented an unacceptable risk. Further confirmation of equivalence between the SL-prepared and manually-prepared MMIX was given by a side-by-side analytical sensitivity comparison (Fig. 9). The Hamilton STARlet method passed acceptance criteria for the method transfer and was introduced into routine use, where it was operated until the retirement of the SARS-CoV-2 screening service. Regulated labs with validated manual workflows that plan to update or improve their assays through automation, may take the method transfer approach if they are substituting a discrete step of an end-to-end assay [29]. In doing so they can dramatically reduce the regulatory burden and the amount of laboratory work required to bring automation improvements online and operational.

With this work, we sought to develop a robotic liquid handling method for MMIX preparation at a high-throughput SARS-CoV-2 screening facility that would provide equivalent results’ quality to a trained human operator. A major aim of the method was to eliminate common errors by laboratory staff when preparing large batches (up to 6,144 reactions) of the PCR MMIX manually (e.g., forgetting to add a reagent, adding the wrong reagent, or adding too much of a reagent). Existing quality and process control methods for the manual MMIX preparation workflows were gravimetric and hence did not support the detection of addition of incorrect liquids of the same density. Similarly, the gravimetric quality control method was insensitive to operators adding incorrect volumes of the critical but relatively small volume of EpiScript. EpiScript is the reverse transcriptase component of the LGC Biosearch Technologies SARS-CoV-2 RT-PCR Test. Without it, cDNA is not transcribed from RNA and hence no cDNA template can be amplified in either controls or samples. This error would only be detected at the end of the process, upon data analysis. While the exact cost per test in terms of reagents is commercially sensitive and cannot be shared here, if one were to assume a reasonable cost of £5 sterling per reaction, errors on this scale become extremely costly (> £30,000), not to mention the cost in terms of sample material, time, and other wasted resources. Another, albeit anecdotal, benefit to automating the preparation of MMIX was a reduction in operator hands-on time. Using the manual workflows, operators were required to manually calibrate pipettes daily, which alongside the gravimetric quality control step could mean that MMIX preparation could take up to 90 min. This procedure favored the production of large batches based on projections. Batch production based on forecasted demand would often lead to the over-preparation of MMIX as preparing an insufficient volume of MMIX would lead to lengthy delays in processing as operators scrambled to prepare more. The automated method on the other hand took approximately 15 min to run end-to-end, which facilitated the preparation of MMIX to order. Further developments and improvements that could be explored include direct integration with Laboratory Information Management Systems (LIMS) and full process automation through collaborative robots. Indeed, laboratories of the future are trending towards standards for integration [30], [31] and self-driving laboratories [32], [33].

It has been highlighted that PCR technologies are not only highly sensitive for detection of targets, but they are also highly sensitive to errors in design and implementation; systemic and random errors can result in false results and irreproducible work [34]. Advocates of automation technology in PCR-based screening workflows highlight the numerous applications of automation during the COVID-19 pandemic, which were often implemented not just for scaling-up operations, but for quality control reasons [4], [35]. Most examples of automated set-up of PCR reactions are only semi-automated as they rely on highly skilled operators to manually prepare a PCR Master Mix from separate reagents before loading this onto an automated liquid handler for dispensing into microtiter plates [6], [7]. Indeed, on a small scale, where the financial risk is relatively low, there is little motivation to invest the significant time and effort required for optimizing a method that requires both a high degree of process flexibility (for varying numbers of samples) and engineering control over the process to prevent deviations. However, looking beyond COVID-19 and the widespread implementation of automated high-throughput PCR screening workflows, previous works have implemented automation to increase throughput and reduce the cost of running high-throughput screening assays manually [36], while others have automated NGS workflows to minimize the potential for human error while using costly enzymatic reagents [37]. A clear application of the process control logic that we have described here is within the field of high-throughput bulk sequencing workflows, where the most expensive components of an assay formulation, by a large margin, tend to be the enzymes used in library preparation. Taken together with other strategies such as miniaturization of assay reactions and in-house manufacture of simple laboratory hardware [38], processes that maximize opportunities for reagent recovery would be highly advantageous for reducing operating costs for sequencing laboratories.

## 5 Conclusion

We hope that by making this work available, it provides an example to the laboratory automation community of the development of liquid handling workflows for MMIX preparation, and the logic that can be used to minimize wastage. The automation of MMIX preparation is important to ensure consistency and quality in high-throughput molecular diagnostic applications. Plans for scaling up operations in response to demand from a “Pathogen X” outbreak [16] should factor in automation, and carefully consider the logic required to ensure demand and quality are both met.

## Funding

The laboratory work was financially supported by the UK Department of Health and Social Care through the Test and Trace program. The laboratory work was conducted at the Rosalind Franklin Laboratory (Royal Leamington Spa, UK).

## Research ethics statement

As all data were obtained from pseudonymized patient samples routinely collected as part of the Test and Trace program, no prospective samples were collected. The use of samples for PCR testing, as described in this work, is covered by the over-arching Privacy Notice issued by the UKHSA with the legal basis for processing such data being:

• GDPR Article 6(1)(e) – the processing is necessary for the performance of its official tasks carried out in the public interest in providing and managing a health service
• GDPR Article 9(2)(h) – the processing is necessary for the management of health/social care systems or services
• GDPR Article 9(2)(i) – the processing is necessary for reasons of public interest in the area of public health
• Data Protection Act 2018 – Schedule 1, Part 1, (2) (2) (f) – health or social care purposes

On the basis of the above and in the context of the public health pandemic response, Tom Fowler (Director of Public Health Testing for COVID-19) of The UK Health Security Agency waived ethical approval for this work. Nicholas Moiseiwitsch (Deputy to Programme Director at Rosalind Franklin Laboratory) of the UK Health Security Agency gave approval for the work to be published.

## CRediT authorship contribution statement

**Giorgio Fedele:** Writing – original draft, Methodology, Investigation, Formal analysis, Conceptualization. **Graham Hill:** Software, Methodology, Conceptualization. **Amelia Sweetford:** Validation, Software, Methodology. **Suki Lee:** Methodology, Conceptualization. **Bobby Yau:** Validation, Resources, Investigation. **Domenico R. Caputo:** Validation, Resources, Investigation. **Denise Grovewood:** Resources. **Rowda Dahir:** Validation, Investigation. **Paula Esquivias Ruiz-Dana:** Validation, Supervision, Project administration, Investigation. **Anika Wisniewska:** Supervision, Project administration. **Anna Di Biase:** Supervision, Project administration. **Miles Gibson:** Supervision, Project administration. **Benita Percival:** Project administration. **Stefan Grujic:** Project administration. **Donald P. Fraser:** Writing – original draft, Supervision, Project administration, Data curation.

## Declaration of competing interest

The authors declare the following financial interests/personal relationships which may be considered as potential competing interests: Giorgio Fedele reports equipment, drugs, or supplies was provided by SPT Labtech Ltd. Giorgio Fedele reports equipment, drugs, or supplies was provided by LGC Biosearch Technologies Inc. Giorgio Fedele reports equipment, drugs, or supplies was provided by BMG LABTECH GmbH. Donald Fraser reports financial support was provided by United Kingdom Department of Health and Social Care. Denise Grovewood reports a relationship with SPT Labtech Ltd that includes: employment. The authors have no further conflicts of interest to declare. If there are other authors, they declare that they have no known competing financial interests or personal relationships that could have appeared to influence the work reported in this paper.

## References

[1] Saiki R.K., Scharf S., Faloona F., Mullis K.B., Horn G.T., Erlich H.A. (1985). Enzymatic amplification of beta-globin genomic sequences and restriction site analysis for diagnosis of sickle cell anemia. Science.

[2] Mullis Kary B. (1990). The unusual origin of the polymerase chain reaction. Sci Am.

[3] Leber Werner, Lammel Oliver, Siebenhofer Andrea, Redlberger-Fritz Monika, Panovska-Griffiths Jasmina, Czypionka Thomas (2021). Comparing the diagnostic accuracy of point-of-care lateral flow antigen testing for SARS-CoV-2 with RT-PCR in primary care (REAP-2). eClinicalMedicine.

[4] Courtney Patrick, Royall Paul G. (2021). Using robotics in laboratories during the COVID-19 outbreak: A review. IEEE Robot Autom Mag.

[5] Van Vooren Steven, Grayson James, Van Ranst Marc, Dequeker Elisabeth, Laenen Lies, Janssen Reile (2022). Reliable and scalable SARS-CoV-2 qPCR testing at a high sample throughput: Lessons learned from the belgian initiative. Life.

[6] Greub Gilbert, Sahli Roland, Brouillet René, Jaton Katia (2016). Ten years of R&D and full automation in molecular diagnosis. Future Microbiol.

[7] Ham Rachel E., Smothers Austin R., King Kylie L., Napolitano Justin M., Swann Theodore J., Pekarek Lesslie G. (2022). Efficient SARS-CoV-2 quantitative reverse transcriptase PCR saliva diagnostic strategy utilizing open-source pipetting robots. JoVE.

[8] Wallace Paul, McCulloch Elaine, Bamford Dennis H., Zuckerman Mark (2021). Encyclopedia of virology.

[9] Guan Xue Li, Chang Dorothy Pei Shan, Mok Zhen Xuan, Lee Bernett (2023). Assessing variations in manual pipetting: An under-investigated requirement of good laboratory practice. J Mass Spectrom Adv Clin Lab.

[10] Roix J., Sudhanva M., Cox T., Curry J., Millican E., Cranston D. (2021).

[11] Figueroa Solange, Freire-Paspuel Byron, Vega-Mariño Patricio, Velez Alberto, Cruz Marilyn, Cardenas Washington B. (2021). High sensitivity-low cost detection of SARS-CoV-2 by two steps end point RT-PCR with agarose gel electrophoresis visualization. Sci Rep.

[12] Silva Júnior José Valter Joaquim, Merchioratto Ingryd, de Oliveira Pablo Sebastian Britto, Rocha Lopes Thaísa Regina, Brites Patrícia Chaves, de Oliveira Elehu Moura (2021). End-point RT-PCR: A potential alternative for diagnosing coronavirus disease 2019 (COVID-19). J Virol Methods.

[13] Cruz-Rangel Armando, Gómez-Romero Laura, Cisneros-Villanueva Mireya, de Anda Jáuregui G., Luna-Pineda Victor, Cedro-Tanda Alberto (2022). End-point RT-PCR based on a conservation landscape for SARS-COV-2 detection. Sci Rep.

[14] WHO (2023).

[15] Cabinet Office, United Kingdom (2022).

[16] Skyle Daniel (2022). WHO Pathogen X conference. Lancet Infect Dis.

[17] Hess J.F., Kohl T.A., Kotrová M., Rönsch K., Paprotka T., Mohr V. (2020). Library preparation for next generation sequencing: A review of automation strategies. Biotechnol Adv.

[18] Holland P.M., Abramson R.D., Watson R., Gelfand D.H. (1991). Detection of specific polymerase chain reaction product by utilizing the 5’—-3’ exonuclease activity of thermus aquaticus DNA polymerase. Proc Natl Acad Sci U S A.

[19] Lu Xiaoyan, Wang Lijuan, Sakthivel Senthilkumar K., Whitaker Brett, Murray Janna, Kamili Shifaq (2020). US CDC real-time reverse transcription PCR panel for detection of severe acute respiratory syndrome coronavirus 2. Emerg Infect Dis.

[20] Ginzinger David G. (2002). Gene quantification using real-time quantitative PCR: An emerging technology hits the mainstream. Exp Hematol.

[21] Ph Eur (2023).

[22] Cabinet Office, United Kingdom (2020).

[23] Food and Drug Administration (2007).

[24] Hess A.S., Shardell M., Johnson J.K., Thom K.A., Strassle P., Netzer G. (2012). Methods and recommendations for evaluating and reporting a new diagnostic test. Eur J Clin Microbiol Infect Dis.

[25] Berkson Joseph (1944). Application of the logistic function to bio-assay. J Amer Statist Assoc.

[26] R Core Team (2023).

[27] Du Hailing, Zhang Yingshuang, Jiang Hongru, Wang Hui (2022). Adsorption of rhodamine b on polyvinyl chloride, polystyrene, and polyethylene terephthalate microplastics in aqueous environments. Environ Technol Innov.

[28] Hawker Charles D. (2017). Nonanalytic laboratory automation: A quarter century of progress. Clin Chem.

[29] Webster Gregory K., Kotts Laila, Maloney Todd D. (2005). Considerations when implementing automated methods into gxp laboratories. JALA: J Assoc Lab Autom.

[30] Bär Henning, Hochstrasser Remo, Papenfub Bernd (2012). SiLA: Basic standards for rapid integration in laboratory automation. J Lab Autom.

[31] Bromig Lukas, Leiter David, Mardale Alexandru-Virgil, von den Eichen Nikolas, Bieringer Emmeran, Weuster-Botz Dirk (2022). The SiLA 2 manager for rapid device integration and workflow automation. SoftwareX.

[32] Marescotti Diego, Narayanamoorthy Chandrasekaran, Bonjour Filipe, Kuwae Ken, Graber Luc, Calvino-Martin Florian (2022). AI-driven laboratory workflows enable operation in the age of social distancing. SLAS Technol.

[33] Abolhasani Milad, Kumacheva Eugenia (2023). The rise of self-driving labs in chemical and materials sciences. Nat Synth.

[34] Taylor Sean C., Nadeau Katia, Abbasi Meysam, Lachance Claude, Nguyen Marie, Fenrich Joshua (2019). The ultimate qPCR experiment: Producing publication quality, reproducible data the first time. Trends Biotechnol.

[35] Jonguitud-Borrego Nestor, Malcı Koray, Anand Mihir, Baluku Erikan, Webb Calum, Liang Lungang (2022). High-throughput and automated screening for COVID-19. Front Med Technol.

[36] Chen Jing, Futran Alan, Crithary Austin, Li Sha, Wolicki Alex, Fogarty Kylie (2021). Leveraging automation toward development of a high-throughput gene expression profiling platform. SLAS Discov.

[37] Santacruz Diana, Enane Francis O., Fundel-Clemens Katrin, Giner Martin, Wolf Gernot, Onstein Svenja (2022). Automation of high-throughput mRNA-seq library preparation: a robust, hands-free and time efficient methodology. SLAS Discov.

[38] Li Haichao, Wu Kun, Ruan Chenchen, Pan Jiao, Wang Yujin, Long Hongan (2019). Cost-reduction strategies in massive genomics experiments. Mar Life Sci Technol.

